# Towards targeting PD-1/PD-L1 axis in breast cancer, pre-clinical data

**DOI:** 10.1186/2051-1426-3-S1-P7

**Published:** 2015-08-14

**Authors:** Hazem Ghebeh, Dilek Colak, Asma Tulbah, Abdullah Alsuliman

**Affiliations:** 1 Stem Cell & Tissue Re-engineering Program, King Faisal Specialist Hospital and & Research Centre, Takhassusi Road, Al-Maathar, Riyadh, Saudi Arabia; 2 Department of Biostatistics, Epidemiology and Scientific Computing, King Faisal Specialist Hospital & Research, Takhassusi Road, Al-Maathar, Riyadh, Saudi Arab; 3 Department of Pathology and Laboratory Medicine, King Faisal Specialist Hospital & Research Centre, Riyadh, Saudi Arab

## 

PD-L1 is a ligand that upon binding to its receptor (PD-1) on T-cells leads to T-cell anergy/and or apoptosis [1,2]. We have shown that PD-L1 is expressed in breast cancer where its expression correlates with estrogen receptor (ER) negativity [3]. To understand the mechanism of the constitutive expression of PD-L1 in tumor cells of ER negative cells we used gene-in and out approach, large scale bioinformatics and immunohistochemistry. We have demonstrated that epithelial to mesenchymal transition (EMT) upregulates PD-L1 expression while cells expressing ER downregulates PD-L1, in parallel with reversal of EMT process. Bioinformatics analysis of gene expression signatures of breast tumors showed a significant correlation between EMT score and PD-L1 mRNA expression. Strikingly, very strong association were found between PD-L1 expression and claudin low breast cancer, a subset of breast cancer known to have high EMT score. In conclusion, we have characterized the expression of PD-L1 in breast cancer and we have demonstrated a strong association between PD-L1 expression, EMT status and claudin-Low breast cancer. Our finding will be essential for choosing the appropriate subset of breast cancer patients that will likely benefit from anti-PD-L1 targeted therapy and understand biological changes upon anti-PD-L1 therapy.
